# A Dynamic System to Control the Entry of Non-Authorized Visitors and Detect Superspreader Farms in Strongly Interconnected Systems

**DOI:** 10.3390/ani14202932

**Published:** 2024-10-11

**Authors:** Oscar Soriano, Laura Batista, Joaquin Morales, Eduardo Quintana, Carlos Piñeiro

**Affiliations:** 1 Animal Data Analytics, SL, 40006 Segovia, Spain; oscar.soriano@ada-animaldata.com (O.S.); joaquin.morales@ada-animaldata.com (J.M.); eduardo.quintana@ada-animaldata.com (E.Q.); 2 Batista & Asociados, Sainte-Julie, QC J3E 0C6, Canada; laurabatistagarcia@gmail.com

**Keywords:** biosecurity, information technology, livestock management

## Abstract

**Simple Summary:**

This paper addresses the need for effective biosecurity due to disease challenges, the emergence and re-emergence of new pathogens, the growing pressure of antibiotic restrictions, and the welfare and sustainability of livestock farming. It introduces the Biorisk® External platform, a cloud-based visitor control system that enhances data management and biosecurity compliance protocols. This system analyzes visitation patterns, revealing trends and categorizing visits by authorization and risk status. This network analysis also allows for the identification of ‘superspreader’ farms that represent a high epidemiological risk. The results advocate for integrating technology into biosecurity protocols to optimize standard operation procedures (SOPs) to improve animal health and mitigate economic losses, highlighting the importance of data-driven decision-making in modern livestock farming.

**Abstract:**

This study explores the critical challenges the livestock sector faces, particularly those related to biosecurity, animal welfare, and antibiotic use restrictions. It highlights the need to implement advanced information and communication technologies to enhance operational sustainability and decision-making. We introduce the Biorisk^®^ External platform, a cloud-based visit control system designed to optimize biosecurity management by accurately tracking visitor activity through QR codes and GPS geolocation. During a 6-month study period from July to December 2023, we analyzed visits to 142 different swine production sites and 30 vehicle movement patterns. The analysis revealed trends in visitation patterns and compliance with biosecurity SOPs. The software categorized visits as authorized (A), not authorized with access (NAWA), and not authorized without access (NAWOA), providing a framework to assess biosecurity risks. Additionally, network analysis identified interconnected farms, which were classified as ‘superspreaders’, highlighting their considerable risk of disease transmission. This study advocates for the integration of digital systems in livestock operations to improve biosecurity measures, facilitate real-time data input, and support informed decision-making. By enhancing biosecurity protocols through technology, the livestock industry can better safeguard animal health, increase operational efficiency, and reduce potential economic losses associated with disease outbreaks.

## 1. Introduction

The agricultural sector is currently facing significant challenges, with health considerations, such as increasing pressure due to antibiotic restrictions and the need to ensure the welfare and sustainability of livestock farming, being of paramount importance. Consequently, the implementation of intelligent biosecurity (IB) is non-negotiable since it is clear, as stated by Capper [1], that we will not move towards a better industry if we do not eliminate the diseases that are causing extreme losses to the industry. Intelligent biosecurity’s goal is to improve animal health, welfare, and productivity through real-time traceability. Intelligent biosecurity gathers and screens information for signs of emerging issues to help anticipate problems through the analysis of collected data to minimize the threat of any future introduction of pathogens. In addition, IB utilizes technology and data-driven methods to monitor, analyze, and optimize different aspects of livestock production, and very importantly, it is an educational tool for all stakeholders in the swine industry, enhancing their understanding of effective biosecurity to change misguided conduct prototypes. The judicious use of information is crucial for making informed decisions that enhance the sustainability of businesses. In this sense, information and communication technologies play a pivotal role in companies’ operations. A Deloitte report [2] described the potential impact of digitalization in meat chain production. In a comparable way, Gebauer et al. [3] emphasized the critical role of IT in boosting company revenues and productivity, while Drnevich and Croson [4] underscored the transformative role of technology in shaping business strategies, fostering novel value propositions, and enhancing the ability of companies to seize emerging opportunities.

Health status heavily influences the continuity of businesses, particularly concerning virus-related diseases [5]. These encompass devastating conditions like African swine fever (ASF) or foot-and-mouth disease (FMD), as well as epidemics such as porcine reproductive and respiratory syndrome (PRRS) or porcine epidemic diarrhea (PED) in swine and the highly pathogenic avian influenza or Newcastle disease in poultry. The impact of PRRS on business profitability has been extensively studied: Holtkamp et al. [6] estimated losses in the American swine industry associated with PRRS at USD 664 million annually, while in Europe, Nieuwenhuis et al. [7] identified economic losses of EUR 126 per sow due to the same disease. More recent work by Nathues et al. [8] reported losses of EUR 126.79 per sow per year and EUR 3.77 per fattening pig.

Moreover, there is growing pressure to restrict antibiotic use and improve animal welfare standards. In this context, biosecurity is a key component of this equation [9]. Historically, surveys have served as the primary method to drive improvements in biosecurity protocols [10,11,12]. Surveys conducted through standardized methods may fall short of addressing the human factor, which introduces a considerable degree of subjectivity. Conclusions drawn from surveys can be influenced by the surveyor’s expertise and the respondents’ subjectivity.

Ensuring compliance with biosecurity protocols presents challenges, including the reliability of information, the scope for data-driven training, the value of information, the necessary infrastructure, and operational ease, which surveys, farm audits, and surveillance cameras cannot entirely tackle. Sensor-based technology emerges as a promising solution for overcoming these challenges [13]. Leveraging information technologies in biosecurity enhances data quantity and quality, shortens response times—crucial for disease control and containment across farms—and reduces human biases, thereby boosting the reliability of conclusions. Consequently, this approach enhances the efficacy of action plans, lowers costs, and increases business profit margins.

External biosecurity focuses on shielding farms from the entry of diseases, a concern increasingly linked to transport, animals, feed, people, or material. The supervision of incoming visits to farms proves crucial in this regard, with the literature underscoring vehicles and individuals as primary sources of pathogen dissemination between farms [14,15,16,17,18,19].

This study establishes a framework for on-farm biosecurity analysis using a cloud-based platform tool. In addition to traditional numerical and descriptive statistical analyses, this study aims to determine patterns of visits to different facilities. This initial investigation highlights trends indicative of deficient farm visit management or non-compliance with biosecurity SOPs. Future research will include a network analysis that will regionally point out farms that require the most surveillance and changes in biosecurity SOPs.

## 2. Materials and Methods

**Figure 1:**
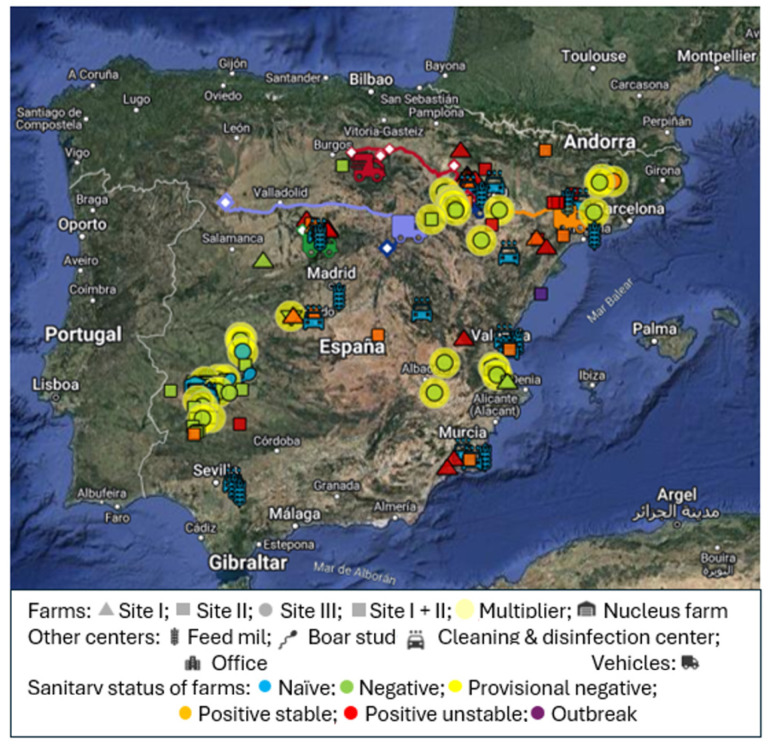
Biorisk^®^ External map of the traced sites. (Figure 1 was obtained from Biorisk^®^ External platform (2024) and reproduced with permission from Animal Data Analytics, SL).

Data collection is a key phase in any information system and has historically posed challenges in the livestock industry, representing one of the most important bottlenecks. While some areas like reproduction, health, and productive performance have generated effective metrics [20,21], the scenario differs significantly in biosecurity compliance. Ensuring adherence to biosecurity protocols represents a unique challenge for each farm or productive system. This issue is particularly challenging regarding visitor management, i.e., individuals and vehicles. Existing registration methods are paper-based, thus preventing non-compliant visits, tracking routes, or foreseeing potential risk issues difficult to accurately monitor any farm or system. With a combination of health, production, and swine industry consultants’ expertise, we have developed an efficient data collection system:

1. Data collection system: Biorisk^®^ External (Animal Data Analytics, SL; Segovia, Spain), a digital visitor control system, is a web application developed in .NET 5 framework by Animal Data Analytics SL in Segovia, Spain, that operates as Software as a Service (SaaS) in the cloud. Users can manually register visits via a cell phone using site-specific QR codes or leverage automatic registration utilizing GPS geolocation data from company or suppliers’ vehicles. Biosecurity rules and downtime movements between sites are customized based on the farm PRRS status [22]. If the farm status changes, the system updates each visitor accordingly with the associated risks, classifying them into three categories: authorized (A), not authorized with access (NAWA), and not authorized without access (NAWOA).
2. Report generation: The application generates reports showing different metrics for the system’s routine management, including alerts, summaries, distributions, outbreak traceability, and its evolution. It can also include a timeline of visits and their category. All this information can be easily exported to perform the network analysis.
3. Period: The system was implemented in two Spanish swine companies with a total of 142 sites and 30 vehicles (Table 1). Initially, a pilot project was conducted from September 2022 to July 2023, involving 30 sites and 4 vehicles. Thereafter, Biorisk^®^ External was implemented in all sites of the two companies and visits to all sites were tracked up to December of 2023. Figure 1 shows a Biorisk^®^ External map of the traced sites.

### 2.1. Statistical Analysis

The methodology for each of the analyses performed is described below. For each of them, R statistical software version 4.1.2 was used.

#### 2.1.1. Visit Evolution Timeline

The visits were studied qualitatively through time series plotting using the ggplot, plotly, and ggthemes libraries. In addition, the empirical 95% confidence interval was calculated using the 0.025 and 0.975 quantiles. This interval was validated using the Dvoretzky–Kiefer–Wolfowitz theorem (DKW inequality):

$$P\left(\underset{x\in R}{\sup}\left|F\left(x\right)-F_{n}\left(x\right)\right|>\varepsilon \right)\leq {2e}^{2n\varepsilon^{2}},\;\;\;\;\;\;\forall \varepsilon >0$$

#### 2.1.2. Comparative Study

The bar and sunburst plots were compared using the ggplot, ggthemes, and plotly libraries. The comparisons included the average number of visits per day of the week, and the type of visit (A, NAWA, and NAWOA). Also, the rate of visits per role (veterinarian, visitor, maintenance, others) and type of visit; the rate of visits per type of vehicle and type of visit; and the rate of visits per day of the week and type of vehicle were plotted. In turn, a quantitative study was carried out by performing a contrast of proportions. To perform this task, we used the R function prop.test which executes the Newcombe–Willson test.

#### 2.1.3. Network Study

A graph is a pair $\left(V,\;E\right)$ where V is a set of generic elements, denoted by nodes, and E⊂V×V is a subset of pairs of elements of V, commonly denoted by a set of edges. An edge $\left(v_{i},\;v_{j}\right)\in \;E$ symbolizes that the node $v_{i}$ is related to the node $v_{j}$; the reciprocal relationship exists if and only if $\left(v_{j},\;v_{i}\right)\in \;E$. Let $V^{\prime }\subset \;V$; the induced subgraph is defined as $\left(V^{\prime },\;E\cap \left(V^{\prime }\times V^{\prime }\right)\right)$.

Given a graph (V, E), it is said to be undirected if for every $\left(v_{i},\;v_{j}\right)\in \;E$, $\left(v_{j},\;v_{i}\right)\in \;E$. On the other hand, it is said to be weighted if there exists an application ϕ: E→ R such that $\phi \left(v_{i},\;v_{j}\right)=\lambda_{i.j}$.

Given a graph (V,E), its adjacency matrix is defined as $A={\left(a_{i,j}\right)}_{1\leq i,j\leq \#V}$, where we have

$$a_{i,j}=\;\left\{\begin{array}{c} 1\;si\;\left(v_{i},v_{j}\right)\in E\\ 0\;si\;\left(v_{i},v_{j}\right)\notin E \end{array}\right.$$

if the graph is not weighted, or

$$a_{i,j}=\;\left\{\begin{array}{c} \phi \left(v_{i},\;v_{j}\right)=\lambda_{i,j}\;si\;\left(v_{i},v_{j}\right)\in E\\ 0\;si\;\left(v_{i},v_{j}\right)\notin E \end{array}\right.$$

if the graph is weighted by an application ϕ.

Given a graph $\left(V,E\right)$ with an adjacency matrix $A=(a_{i,j})$, the *degree* of a vertex $v_{i}\in \;V$ is defined as

$$\deg\left(v_{i}\right)=\sum_{j=0}^{\#V}a_{i,j}$$

Given a graph $\left(V,\;E\right)$ and $v_{i},\;v_{j}\in \;V$, these are connected if $v_{i}=v_{j}$, $\left(v_{i},\;v_{j}\right)\in \;E$ or if there is a path connecting $v_{i}$ and $v_{j}$. The connection defines an equivalence relation in which the subgraphs induced by such equivalence classes are known as connected components associated with the graphs. A graph is a disjoint union of all its connected components.

Let $\left(V,E\right)$ a graph, two vertices $v_{i},v_{j}$ are connected by a path if there exist vertices $v_{k_{1}},\ldots v_{k_{m}}\in V$ such that $\left(v_{i},v_{k_{1}}\right),\left(v_{k_{1}},v_{k_{2}}\right),\ldots ,\left(v_{k_{m-1}},v_{k_{m}}\right),\left(v_{k_{m}},v_{j}\right)\in E$. A graph is said to be strongly connected if there is a path connecting two pairs of vertices.

Being $\sigma \left(A\right)$ the spectrum (i.e., the set of eigenvalues) of the adjacency matrix, and supposing that it is non-negative irreducible, since the graph is strongly connected, then, by the Perron–Frobenius theorem, it exists a strictly dominant eigenvalue $\hat{\lambda }\in \sigma \left(A\right)$ and an eigenvector $x_{\hat{\lambda }}$ whose coordinates are all positive. The eigenvalue centrality associated with node $v_{i}$ is the i-th coordinate of $x_{\hat{\lambda }}$. It must be considered that eigenvalue centrality is a qualitative measure of the importance of a vertex over a strongly connected graph, independently of the scale. Given an undirected graph $\left(V,\;E\right)$ with adjacency matrix A, its lagrangian matrix is defined as

$$L=A-\left(\deg\left(v_{1}\right),\;\ldots ,\deg\left(v_{m}\right)\right)\cdot \;I$$

The subspace of eigenvectors associated with the null eigenvalue of the lagrangian matrix has a basis of vectors $\beta =\;<x_{1},\;\ldots ,x_{n}>$ satisfying the following properties:

- The vectors $x_{i}$ have only ones or zeros for their coordinates;
- If the vector $x_{i}$ has a 1 in the j-th coordinate, then the rest of the vectors in the basis have zeros in that coordinate.

Each of these vectors is in correspondence with one of the connected components, which are defined as the subgraphs generated by the vertex set associated with the non-zero coordinates of the vectors (i.e., if $x_{1}=\left(1,\;1,\;1,\;0,\;0\right)$ is one of the above vectors, then the graph generated by the vertex set $\left\{v_{1},\;v_{2},\;v_{3}\right\}$ would be one of the connected components).

It should be noted that, by definition, the connected components of a graph are themselves strongly connected graphs; therefore, it makes sense to speak of eigenvalue centrality in each of these. In the context of this study, set V consists of the sites monitored by Biorisk^®^ External (Animal Data Analytics, SL; Segovia, Spain). Two sites A, B∈ V, are considered epidemiologically related if a vehicle has gone from A to B. This fact will be expressed as $\left(A,\;B\right)\in \;E\subset \;V\times V$.

Given two epidemiologically connected sites, we define the weighting on $\;\phi \left(A,B\right)$ as the number of times a vehicle has gone from A to B plus the number of times that the vehicle has gone from B to A. The adjacency matrix associated with this problem is symmetric; therefore, the graph to be studied is undirected. Once the adjacency matrix has been composed, the lagrangian matrix is calculated to obtain the connected components of the network. In the physical sense, each connected component is understood as a zone of interaction between sites.

Finally, the eigenvalue centrality is calculated for each of the connected components. A superspreader within a connected component will be the farm or farms with the highest eigenvalue centrality, which translates as those farms that epidemiologically interact the most with the others.

## 3. Results

- Visit analysis

The number of visits was calculated by detecting the vehicle plate number, the visited site, and visit date. The dispersion of daily visits was studied with a 95% empirical confidence interval, which resulted in 1 to 60 visits in one day. In addition, this interval was contrasted using the DKW inequality, which found that the probability of deviation more than one visit from the theoretical confidence interval is lower or equal $2\cdot \exp\left(-2\cdot 7812\cdot 1\right)<<0.0001$.

**Figure 2:**
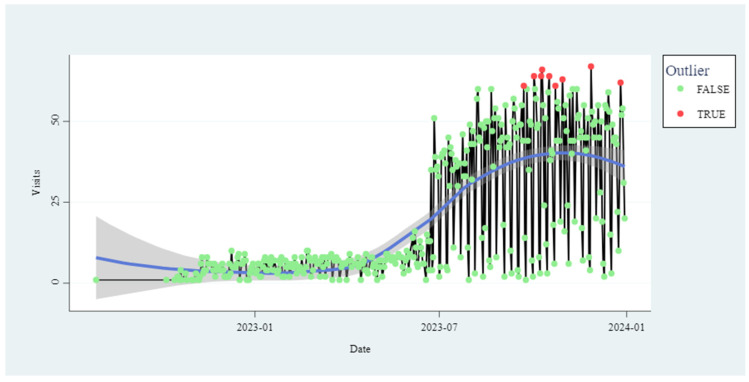
Visit timeline (number).

Based on these results, visits were plotted on a daily time series. Figure 2 shows the days with unusual personnel flow, and its trend was calculated by local regression. This analysis also shows, first, an increased visit pattern starting in July of 2023, second, unusually high activity compared to the pre-established level from the end of September to the beginning of November, and third, the sigmoid curve with stabilization at the end of the study period. After the pilot project, the system was implemented in all the company sites as shown in the stabilization curve to the end of December 2023 (Figure 2).

**Figure 3:**
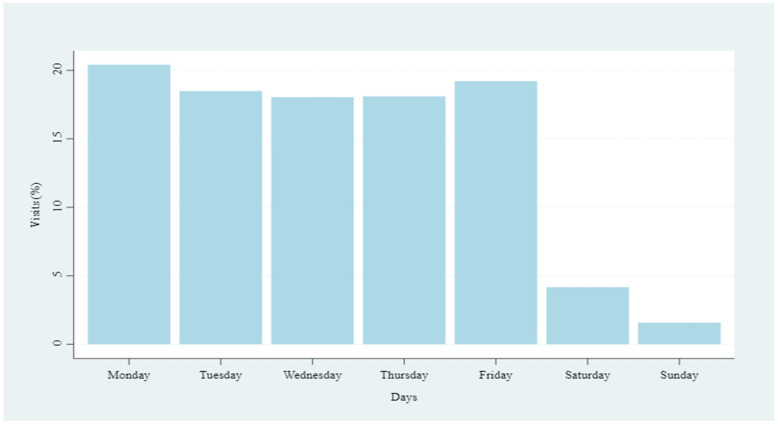
Percentage distribution per weekday.

Visits were also analyzed by date and weekday (Table 2 and Figure 3).

The Newcombe–Willson test results show a lower number of visits on Saturdays and Sundays (*p* << 0.05), in agreement with regular farm standards; Mondays have the highest visit percentage l (*p* << 0.05), and Fridays have significantly more visits than Tuesdays, Wednesdays, and Thursdays (*p* = 0.031). No significant differences were detected between Tuesday, Wednesday, and Thursday (*p* = 0.549).

**Figure 4:**
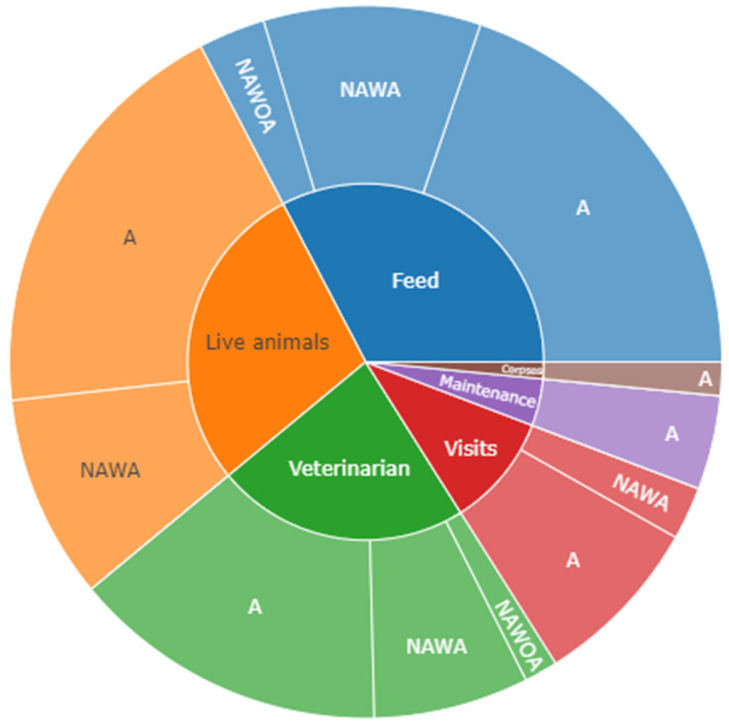
Visit sunburst chart by categories.

**Figure 5:**
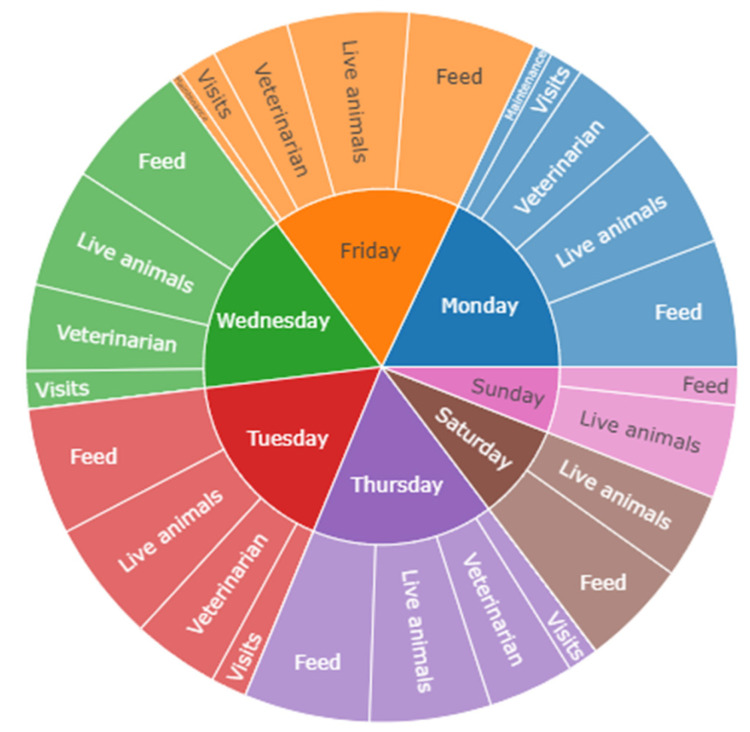
Weekday and category sunburst chart.

Figure 4 shows a sunburst chart of visit distribution by category: authorized (A), not authorized with access (NAWA), and not authorized without access (NAWOA). The results of the Newcombe–Willson test show that category A visits, which mostly consist of veterinarian visits, live animal introduction, and feed delivery (*p* << 0.05), are the principal category (*p* << 0.05), as can be seen in Figure 5.

The implementation of Biorisk^®^ External (Animal Data Analytics, SL; Segovia, Spain) resulted in a significant improvement in data collection and its characterization. As shown in Figure 5, the digitalization of visitor logs facilitated timely and accurate tracking of biosecurity protocols and their compliance, therefore reducing the risk of pathogen introduction into a farm.

- Route Analysis

**Figure 6:**
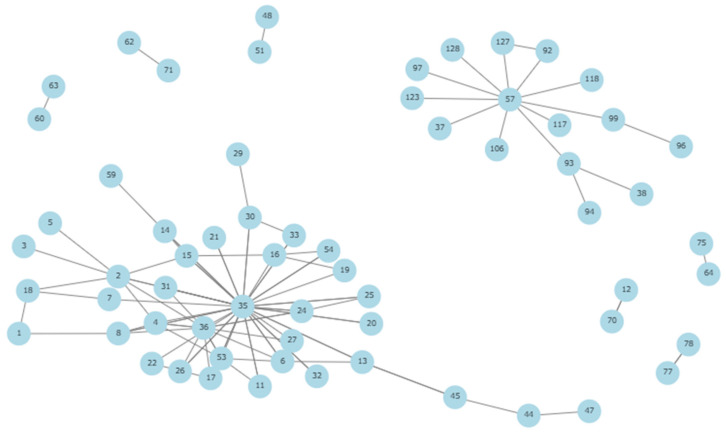
Graph route epidemiological relationships between sites (Each sphere represents a site and its identification number).

**Figure 7:**
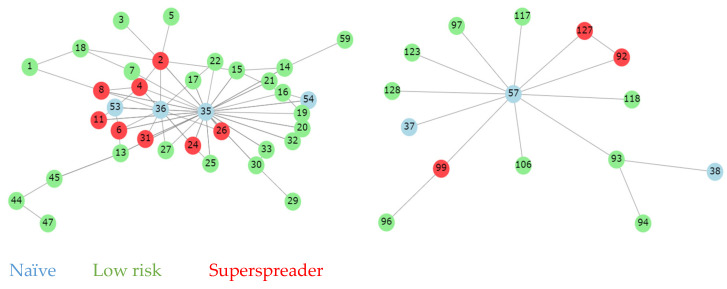
Qualitative representation of massive components (Each sphere represents a site and its identification number).

In this study, a route was defined as a pair of time-ordered visits between two different sites performed by the same single vehicle on the same day. Figure 6 shows that the company’s sites were distributed in eight connected groups. Since each component is strongly connected, by construction, we calculated the eigenvector centrality for each site. As mentioned before, eigenvector centrality indicates the influence of a site within its group, the greater it is, the more likely that it will be affected by a new epidemiological relationship. As shown in Figure 7, a farm was considered a superspreader if its centrality was above the third quantile of its group, therefore representing a higher risk of disease dissemination.

## 4. Discussion

As the livestock production sector faces health, sustainability, and regulatory compliance pressures, integrating advanced data management systems emerges as an imperative strategy to promote animal welfare and efficiency. Traditional paper-based systems have long been identified as inadequate due to human error and inefficiency in capturing real-time data. Consequently, the findings of this study highlight the increasing role of incorporating information and communication technologies to reinforce biosecurity protocols within the sector. The use of Biorisk^®^ External (Animal Data Analytics, SL; Segovia, Spain) improves the accuracy and the quality of people and vehicle movement data management. The digitalization of visitor logs not only facilitates data collection, but it also allows for an immediate response to biosecurity threats. The transition to a cloud-based framework enables stakeholders to make objective data-driven assessments and decisions which are necessary in an era characterized by rapid technological advancement.

Categorizing visits provides a framework to evaluate compliance and determine areas which require investigation and intervention. Furthermore, the analysis of visitor patterns reveals crucial insights into operational workflows and SOP compliance in livestock-producing companies. Our results support the studies of [9,10,11], who concluded that human behavior plays a crucial role in the effectiveness of biosecurity measures. Therefore, the continuous training and education of personnel becomes essential in minimizing the risks associated with authorized and non-authorized visits. Additionally, the network analysis of epidemiological connections among farms developed in our study introduces a novel approach to understanding the dynamics of disease transmission pathways. Identifying farms as ‘superspreaders’ provides actionable intelligence for prioritizing resources and interventions. Previous studies have shown the importance of minimizing contact between farms to prevent the spread of diseases [14,15]. This innovative epidemiological mapping of relationships allows the establishment of biosecurity buffer zones and comprehensive vehicle movement protocols to understand and avoid potential lateral pathogen transmission.

New mathematical models based on graphs and dynamic systems have made great contributions to epidemiological studies; however, some generated incorrect conclusions. By transforming the adjacence matrix into a generalized symmetric adjacence matrix and calculating the eigenvalue centrality for each connected component in the system, we avoid incorrect conclusions, thus accurately identifying the ’superspreader’ farms for every group of epidemiological-related sites.

Integrating quantitative data and qualitative observations goes beyond theoretical information, thus allowing farm managers to implement accurate biosecurity interventions, respond proactively to emerging threats, and engage in strategic planning to mitigate risks. The capacity to generate timely reports and alerts within the Biorisk^®^ External system (Animal Data Analytics, SL; Segovia, Spain) enhances operational alertness, allowing for real-time decision-making in a permanently changing environment. In addition, this system allows us to understand the high impact of continuous education on biosecurity decisions related to livestock disease dynamics [23].

The next steps in our project will include other areas of farm management, such as slurry and mortality management, as the present management practices pose significant pathogen transmission risks. Therefore, the traceability of vehicles involved in these tasks could also be highly relevant. Additionally, it is important to assess how the biosecurity information and protocols provided by each system affect compliance with effective biosecurity regulations. As Bucini et al. [23] noted, individual risk perception influences adherence to these regulations. Thus, risk characterization based on objective data can influence behavioral changes that enhance overall farm biosecurity.

In summary, this research highlights the transformative potential of integrating advanced data management systems in the livestock sector. By enhancing biosecurity protocols through informed decision-making, the industry can better protect animal health, ensure sustainability, and mitigate the significant economic losses associated with disease outbreaks. The findings of this study advocate for a change in our biosecurity mindset, emphasizing the critical combination of technology, data analytics, and proactive operational strategies in overcoming the multiple challenges posed by modern livestock farming.

## 5. Conclusions

This study highlighted the necessity of transitioning conventional paper-based recording to digital frameworks to ensure reliable data capture. The successful implementation of Biorisk^®^ External (Animal Data Analytics, SL; Segovia, Spain) optimizes the combination of advanced information and communication technologies that can significantly improve biosecurity protocols. This system can be used in any livestock sector, thus shielding operations from the risk of pathogen introduction. Also, farm managers will be able to make informed decisions to optimize resource allocation and operational practices by utilizing accurate and real-time data according to visitor patterns in their system. This effective data management will enhance their ability to quickly respond to potential biosecurity threats, reducing economic losses and protecting livestock welfare. Also, the results highlight the critical need for continuous farm personnel education and training regarding biosecurity measures. Mitigating human-related risks associated with unauthorized visits is essential, suggesting that educational programs should be integrated into farm management practices to foster a culture of compliance and awareness. New knowledge on how to identify ‘superspreader’ farms underlines the importance of recognizing interconnectedness among farms. Knowledge that enables stakeholders to prioritize monitoring and intervention strategies for specific facilities enhances the overall resilience of livestock operations against infectious diseases. Finally, our results advocate for developing strategies that support adopting innovative technologies in agriculture.

Future research should continue to explore the positive long-term impact of implementing these technological tools on both animal health improvement and economic performance, fostering a deeper understanding of the interaction between technology, compliance, and sustainability of biosecurity in the livestock industry.

## Figures and Tables

**Table 1:** Site and vehicle categories.

Category	Number
Cleaning and disinfection centers	14
Feed mills	10
Sites I	24
Sites I and II	63
Sites III	24
Boar studs	2
Nucleus farms	3
Multipliers farms	2
Vehicles (no categorization)	30

**Table 2:** Days with unusually high visit activity.

Date	Number of Visits
22 September 2023	61
02 October 2023	64
09 October 2023	64
10 October 2023	66
17 October 2023	64
23 October 2023	61
30 October 2023	63
27 November 2023	67
26 December 2023	62

## Data Availability

The data presented in this study are available on request from the corresponding author. The data are not publicly available due to confidentiality agreements with the pig companies involved in the study.
